# Synthesis of Hybrid Epoxy Methacrylate Resin Based on Diglycidyl Ethers and Coatings Preparation via Cationic and Free-Radical Photopolymerization

**DOI:** 10.3390/ijms232415592

**Published:** 2022-12-09

**Authors:** Paulina Bednarczyk, Izabela Irska, Konrad Gziut, Karolina Mozelewska, Paula Ossowicz-Rupniewska

**Affiliations:** 1 Department of Chemical Organic Technology and Polymeric Materials, Faculty of Chemical Technology and Engineering, West Pomeranian University of Technology in Szczecin, Piastów Ave. 42, 71-065 Szczecin, Poland; 2 Department of Materials Technology, Faculty of Mechanical Engineering and Mechatronics, West Pomeranian University of Technology in Szczecin, Piastów 19 Avenue, 70-310 Szczecin, Poland

**Keywords:** epoxy methacrylates, diglycidyl ethers, coatings, photopolymerization

## Abstract

A series of difunctional epoxy methacrylate resins (EAs) containing at least one epoxy and at least one methacrylate group were synthesized by means of an addition reaction between epoxy-terminated diglycidyl ethers and methacrylic acid. In order to investigate the impact of polymer architecture on the course of addition reactions and further coating properties, several different types of diglycidyl ethers, i.e., linear, containing aliphatic or aromatic rings, with a short or polymeric backbone, were employed in the synthesis. The carboxyl–epoxide addition esterification reactions have been found to, in a relatively straightforward manner, control the extent of acrylation depending on the substrate feed ratio and reaction time. The structure of obtained pre-polymers was evaluated by FT-IR and NMR methods. At the same time, the extent of addition reactions was validated via quantitative analysis, including non-volatile matter content (NV), acid value (PAVs), and epoxy equivalent value (EE) analysis. The modification was carried out in a manner likely to create a compound with one epoxy and one carbon–carbon pendant group. Hence, due to the presence of both functionalities, it is possible to crosslink compositions based on synthesized EAs via two distinct mechanisms: (i) cationic polymerization or (ii) free-radical polymerization. Synthesized epoxy methacrylate pre-polymers were further employed for use in formulate photocurable coating compositions by the cationic or radical process. Furthermore, the photopolymerization behavior and properties of cured coatings were explored regarding some structural factors and parameters. The investigated polymeric materials cure in a short time to obtain coatings with good properties, which is why they can be successfully used to produce protective and decorative coatings for many industries.

## 1. Introduction

Light-induced polymerization is used primarily for the production of coatings, adhesives, printing inks, dental fillings, and hydrogels, as well as 3D-printed elements [1,2,3,4,5,6,7]. Polymer materials that are obtained with the use of electromagnetic radiation unquestionably have many advantages. Among these is the ability to achieve high polymerization rates, which is due to the extremely rapid formation of active centers (radicals or ions) initiating the reaction. Moreover, it is possible to carry out the process at room temperature or lower, on heat-sensitive substrates, in order to use compositions that do not contain organic solvents and to reduce energy consumption [8,9,10]. Additionally, photopolymerization is limited almost exclusively to the irradiated areas, which allows the production of materials of various shapes by simple masking [11]. This method is considered pro-ecological, and the mentioned advantages give it a great advantage over thermally initiated polymerization.

There are two main types of photopolymerization, depending on the type of reactive particles formed during the decay of the photoinitiator [12]: radical photopolymerization or ionic (cationic) photopolymerization. The first is used for (meth)acrylates and unsaturated polyesters. According to the second mechanism, epoxy monomers, oxetanes, and vinyl ethers are photopolymerized. The course of radical photopolymerization is adversely affected by oxygen. It can suppress the initiator excited states, significantly reducing the efficiency of initiation. In addition, oxygen reacts abruptly with radicals on the carbon atom (radicals that promote or arise from the decay of the initiator) to form peroxygen radicals. These processes can significantly slow down polymerization or even stop it completely [8]. In contrast, the course of cationic photopolymerization remains intact in the presence of oxygen. A characteristic feature of this mechanism is the possibility of obtaining a large increase in polymer conversion even after removing the radiation source [13].

The chemical structure of UV-curable resin can be roughly divided into four groups: epoxy acrylates, polyester acrylates, urethane acrylates, and silicone acrylates. Among these, the epoxy acrylates, so-called vinyl ester resins (VERs), have been found especially desirable in applications such as UV-curable paints and varnishes [14]. Conventional industrially significant VERs are synthesized via the addition of unsaturated carboxylic acids, in particular (meth)acrylic to the oxirane groups of diglycidyl ether of bisphenol A (DGEBA) or bisphenol A diepoxy resins [15,16]. The combination of both epoxy backbone and reactive unsaturated end-groups of the VERs offers unique properties such as good mechanical and thermal performance, excellent chemical resistance, adhesion, and fast curing [17,18].

In contrast to the numerous papers on VERs, only a few works dealing with multi-functional monomers/resins having two types of photopolymerizable groups in the structure have appeared until now [19,20,21,22]. In principle, hybrid EAs with both epoxy and vinyl end groups were designed to combine UV- and thermal-curing functionalities. Hexion Inc. commercialized such compounds under the trade name Epon. One of the most recognized resins in their offer, called Epon 8111 [23], is a product based on bisphenol A epoxy resin and acrylate. Hexion introduced the latter as low-viscosity resin, which can be employed in rapid setting adhesives, sealing compounds, and wear-resistant coating systems [24]. 

It cannot be overlooked that EA pre-polymers with both epoxy and vinyl end-groups can polymerize via two different polymerization mechanisms—cationic or radical [1,10,25,26,27,28]. Using a suitable photoinitiator, the photopolymerization mechanism can obtain polymers of various structures, types, and properties from the same pre-polymer. Applying a radical photoinitiator allows the formation of a polymer chain that is terminated with unreacted epoxy groups, while applying a cationic photoinitiator results in a structure reach in unsaturated carbon–carbon double bonds. In this context, hybrid monomers/pre-polymers have been receiving more and more attention from the scientific community in the last few years. 

Our previous study [27] presented a series of novel trifunctional epoxy (meth)acrylate resins (EAs) containing at least one epoxy group and at least one acrylate group and were obtained via the addition of (meth)acrylic acid to the triglycidyl ether of trimethylolethane (TMETGE). The present work focuses on synthesizing epoxy methacrylate resins (EAs) based on low-viscous diglycidyl ethers with different chain architectures. It can be considered a logical continuation of earlier work. Herein, the preparation of several epoxy–methacrylate pre-polymers based on bisphenol A diglycidyl ether (DGEBA), cyclohexane dimethanol diglycidyl ether (CHDMDE), neopentyl glycol diglycidyl ether (NPDE), 1,6-Hexanediol diglycidyl ether (HDE), poly (propylene glycol) diglycidyl ether (PDODE), and polytetrahydrofurane diglycidyl ether (PTMODE) is reported. The obtained pre-polymers were studied concerning their chemical structure (FT-IR and NMR spectroscopy) and via quantitative analysis (NV, PAVs, EE, and viscosity). The synthesized resins were investigated as formulations with a cationic or radical photoinitiator. After making the polymeric film, the UV-curing process was monitored using photo-differential scanning calorimetry (photo-DSC), and the properties of cured coatings were investigated.

## 2. Results and Discussion

### 2.1. The Pre-Polymers Synthesis and Characterization

First, the diglycidyl ethers were modified with methacrylic acid, and then the reaction products were used to produce varnish coatings. The as-synthesized products were characterized in terms of non-volatile-matter content (NV), partial acid numbers (PAVs), MAA conversion (MAAC), epoxy equivalent (EE), conversion of epoxy group (EGC), and viscosity (η). The basic physicochemical properties are presented in Table 1. From the collected results, one can deduce that the rate of addition reactions differs significantly for the tested systems. The obtained products differed in the conversion of the acid, from 77% for modified poly (propylene glycol) diglycidyl ether (PDODE) up to nearly 98% for product based on 1,6-hexane diglycidyl ether (HDE). This result can be ascribed to differences in chain architecture, reactivity, viscosity, molecular weight, and molecular mobility of applied diglycidyl ethers. All of these factors can affect the process and the final degree of MAA or EG conversion. Certainly, at this stage, it is not possible to specify a single factor that determines the reaction rate. For example, it can be noted that at the same reaction time the degree of MAA conversion is almost equally high for both DGEBA-MAA and HDE-MAA systems, which are characterized by the highest and the lowest values of viscosity among the tested systems. In addition, the reaction rate cannot be explicitly related to the molecular weight, since it appears that polymeric PTMODE with a molecular weight of 780 g/mol reacts faster with MAA acid as compared to polymeric PDODE with a molecular weight of 380 g/mol. The obtained products (apart from the modified DGEBA) are characterized by a relatively low viscosity of 100–500 Pa·s, which allows them to be successfully used as the main component of the UV-curable varnish compositions.

**Figure 1:**
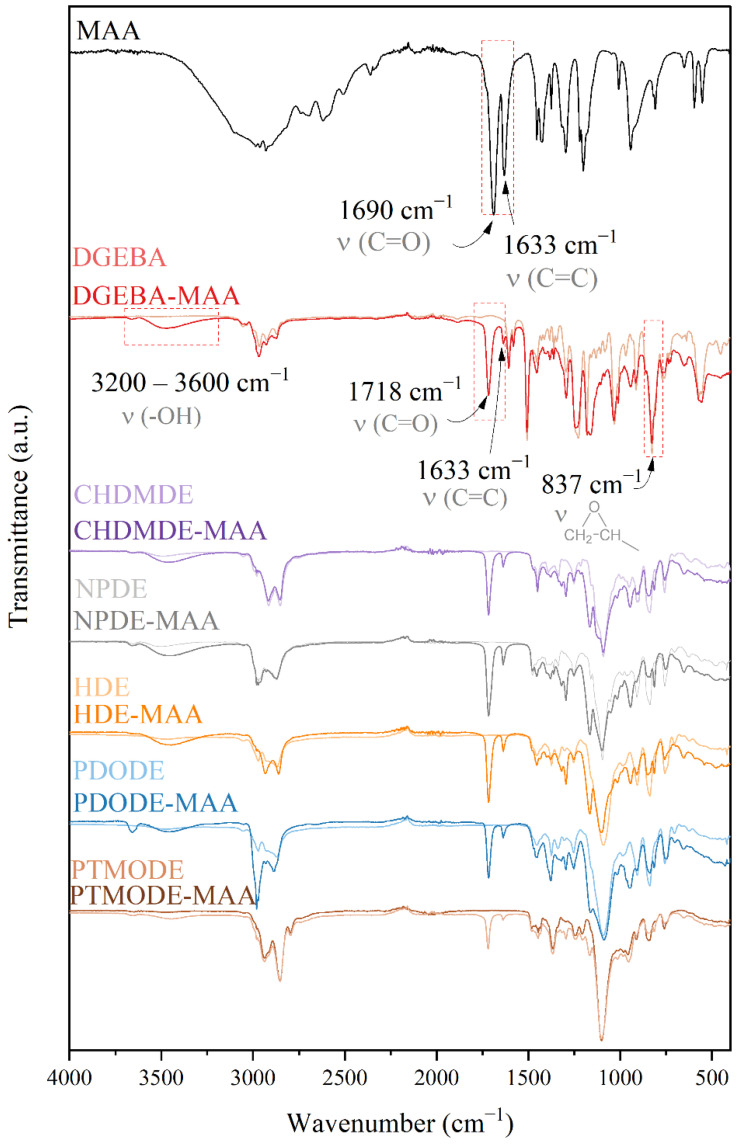
FT-IR spectra of reaction substrates and epoxy methacrylated pre-polymers.

The chemical structure of MAA, diglycidyl ethers, and post-reaction compounds were investigated by means of FT-IR spectroscopy (Figure 1). The absorption bands corresponding to the vibrations of both carboxyl and epoxy groups are of particular interest to investigate the progress of addition reaction in the studied system. The sharp peaks at 1690 cm^−1^ and 1633 cm^−1^ at MAA can be attributed to C=O stretching and C=C vibrations, respectively. As shown in Figure 1, signals characteristic for both C=C and C=O are also evident at pre-polymers spectra. However, a band characteristic of C=O vibrations appears at a higher wavenumber concerning C=O vibrations in MAA. An additional absorption peak develops at a wavelength of 1718 cm^−1^ at the DGEBA-MAA spectrum. This phenomenon can be reasonably ascribed to the formation of ester bonds due to the reaction of carboxyl and epoxide groups of MAA and DGEBA, respectively [29]. A similar effect can be observed in other diglycidyl ether-based systems reported in the present work. As it can be seen from reaction course, one can expect that in the transesterification reaction between diglycidyl ether and methacrylic acid the product may be a mixture of (i) neat diglycidyl ether, (ii) a hybrid compound having both epoxy and vinyl end-groups, and (iii) a compound with vinyl-end groups. Thus, apart from the changes in the C=O and C=C stretching region, one can observe significant changes in the intensity of the IR absorption bands arising from stretching vibrations of oxirane groups at ~3057 cm^−1^ (-C-H), ~914 cm^−1^ (C-O), and ~825 cm^−1^ (C-O-C), which decreased substantially (Figure 1). In contrast, the intensity of the broadband that spans from 3600 to 3200 cm^−1^ increased after modification. The above changes confirm unambiguously that the ring-opening reactions proceed with the formation of additional -O-H groups. In all cases, the spectra were consistent with the expected structures.

**Figure 2:**
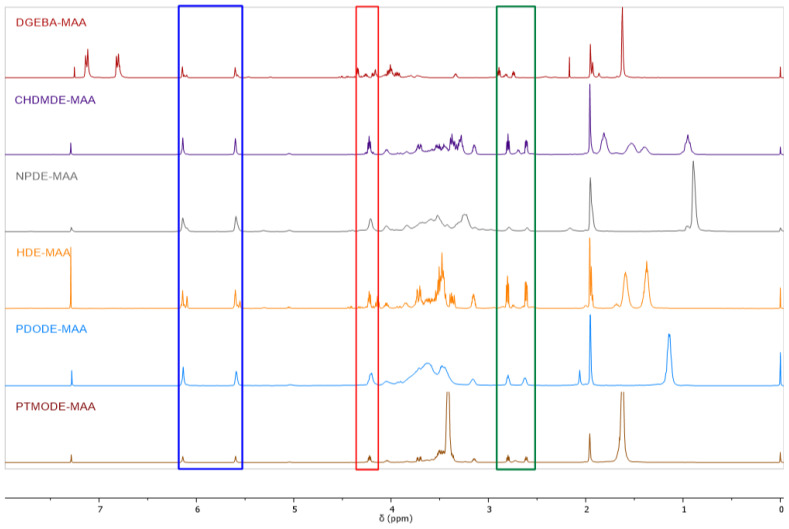
^1^H NMR spectra of epoxy methacrylated pre-polymers.

Moreover, the identity of the compounds obtained was confirmed by analyzing the ^1^H NMR spectra. On this basis, the composition of the post-reaction mixture was also determined, i.e., the share of the unreacted substrate (A) and products (B) and (C), respectively. Figure 2 shows a summary of the ^1^H NMR spectra of all obtained products, respectively, while Table 2 shows the shares of products in the reaction mixture. The number of polymer segments (n) was also assessed by this method. In the comparison of ^1^H NMR spectra, the red square marks the range in which the signal confirming the transesterification reaction occurs (4.18–4.20 ppm). There is a signal from the protons of the CH(OH) group. In addition, the blue color marks the signals from the protons of the CH_2_=CH methacrylic group, while the green color marks the signals from the epoxide group. Based on the analysis of the integration of relevant signals, the shares of individual reaction products in relation to each other were also determined. The ^1^H and ^13^C NMR spectra of all reaction mixtures are summarized in the Supplementary Materials (Figures S1–S14). It has been shown that the desired product (B) is formed in all cases. However, the reactivity depends on the diglycidyl ether used. In the reaction mixture spectra DGEBA-MAA, HDE-MAA, and PTMODE-MAA, signals from the unreacted substrate—diglycidyl ether—are also visible. In the first two cases, above 30% of unreacted substrate is still observed, and in the third one, about 18%. The other reactions show greater reactivity of the diglycidyl ethers used, and this method’s amount of unreacted substrate is below the quantification. Molar ratios of individual products are shown in Table 2. In the case of CHDMDE-MAA and PTMODE-MAA, only a hybrid product containing epoxy and vinyl terminal groups is formed. In the remaining cases, the formation of B and C products is observed, and the highest proportion of the compound with vinyl end groups was observed for NPDE-MAA. Interestingly, by comparing the data obtained by ^1^H NMR quantitative analysis with the results collected in Table 1, one can note that in systems characterized by a high degree of MAAC, i.e., DGEBA (MAAC~94%) and HDE (MAAC~98%), nearly equimolar mixture of A, B, and C products was obtained.

### 2.2. The Photocuring of the Epoxy Methacrylate Pre-Polymers

**Figure 3:**
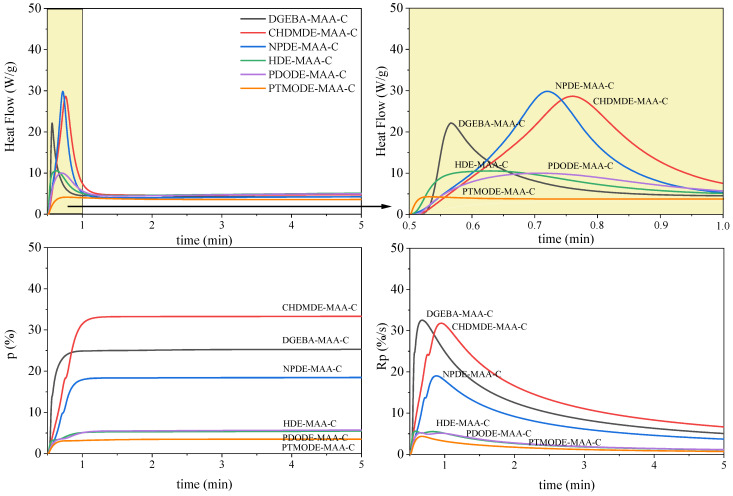
Photo-DSC exotherms for the cationic (C) photopolymerization of epoxy methacrylate formulations (diglycidyl ether-MAA).

**Figure 4:**
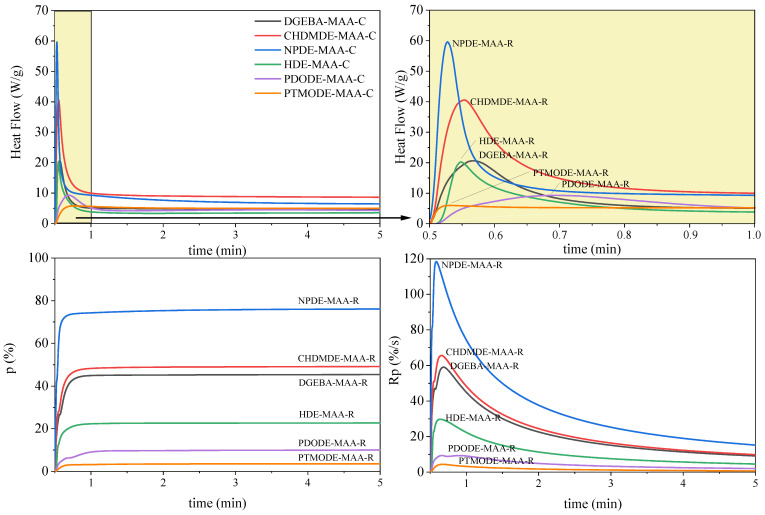
Photo-DSC exotherms for the radical (R) photopolymerization of epoxy methacrylate formulations (diglycidyl ether-MAA).

It is well known that epoxides and acrylates do not polymerize by the same polymerization mechanism. Hence, polyether-type polymer chains with pending methacrylate groups were formed when cationic photoinitiators were applied. In contrast, a polymethacrylate backbone with pending epoxy groups was formed in the case of free-radical polymerization [25]. The research presents the influence of different chain architectures of synthesized epoxy methacrylate resins (EAs) based on low-viscous diglycidyl ethers in the course of the photocuring process according to the cationic and radical mechanisms. In the present study, epoxy methacrylate pre-polymers based on bisphenol A diglycidyl ether (DGEBA), cyclohexane dimethanol diglycidyl ether (CHDMDE), neopentyl glycol diglycidyl ether (NPDE), 1,6-Hexanediol diglycidyl ether (HDE), poly (propylene glycol) diglycidyl ether (PDODE), and polytetrahydrofurane diglycidyl ether (PTMODE) were investigated. Figure 3 and Figure 4 show the kinetics of photopolymerization, taking into account photo-DSC exotherms (heat flow), the degree of conversion (p) of epoxy or methacrylate groups, and the polymerization rate (Rp) for the cationic or radical photopolymerization of the epoxy methacrylate systems containing different photoinitiators (C—cationic or R—radical; Table 3).

The course of the cationic photopolymerization process of the examined EAs systems is quite diverse (Figure 3). Epoxy methacrylate pre-polymers with shorter chain lengths and features of chemical structures such as aromatic, cycloaliphatic, or some branched structures, i.e., DGEBA, CHDMDE, and NPDE, polymerize with a heat flow observed in the form of a sharp peak. In contrast, the heat flow of the photopolymerization process of epoxy methacrylates based on diglycidyl ethers containing a linear carbon chain structure, i.e., HDE, PDODE, and PTMODE, is mild. Hence, the fastest polymerizing systems are EAs based on DGEBA, CHDMDE, and NPDE, with a polymerization rate above 15 s^−1^. The shorter carbon chains of the pre-polymers will likely result in increased mobility of the end groups involved in the cationic polymerization, thus decreasing the limitations of the reaction–diffusion. The fully aliphatic and linear EAs have longer carbon chains, which provide steric hindrance and lead to far proximity of reactive groups. The highest conversion of epoxy groups (p) of the obtained pre-polymers was achieved in the order CHDMDE-MAA > DGEBA-MAA > NPDE-MAA > HDE-MAA > PDODE-MAA > PTMODE-MAA. A similar course of the reaction was observed in the case of studying the polymerization of these systems according to the radical mechanism (Figure 4). The difference, however, is the rate of reaching the maximum enthalpy of the reaction, which, in the case of the radical process, was much higher and achieved in a shorter time. Thus, the radical polymerization rate and the ability to form a polymethacrylate backbone with pending epoxy groups is substantially higher than that of the cationic one. Such conclusions were also described in our previous study [27]. In this case, epoxy methacrylates with a branched carbon chain (based on neopentyl glycol diglycidyl ether (NPDE)) further a system containing a cyclic ring (based on cyclohexane dimethanol diglycidyl ether (CHDMDE)), and EA containing aromatic rings (based on bisphenol A diglycidyl ether (DGEBA)) polymerized the fastest and achieved the highest conversion of the unsaturated double bond of methacrylate groups. The radical process achieved a higher degree of conversion of the reactive groups than the cationic process. NPDE-MAA was characterized by the highest conversion of unsaturated bonds of almost 80%, while as a result of polymerization, according to the cationic mechanism, the conversion was less than 20%.

In order to study the influence of different chain architectures of synthesized epoxy methacrylate resins and the kinetics of photopolymerization on the cured films, the basic utilitarian properties of cured coatings were determined and are presented in Table 4, taking into account the photopolymerization mechanism (C—cationic, R—radical). Despite the different kinetic parameters, including the conversion rate of the reactive groups of the obtained pre-polymers, the tack-free time of the coatings is quite similar and amounts to about 90 s. However, we can distinguish the DGEBA-MAA coating, characterized by the shortest tack-free time, which was 15 and 9 s for the cationic and radical process, respectively, and CHDMDE-MAA, respectively, which was 21 s for the radical process. Interestingly, despite the commonly known oxygen inhibition effect in the radical process, it was in this case that the coatings with shorter surface dry time were obtained (for DGEBA-MAA and CHDMDE-MAA). This type of phenomenon is reflected in the results of photopolymerization kinetics, which had a faster course in the case of radical polymerization as compared to cationic polymerization. EAs obtained on the basis of DGEBA and CHDMDE were also characterized by the highest hardness of coatings, both those obtained in the cationic and the free-radical process, which may result from the characteristic structure of the carbon chain containing aromatic or cyclic rings, which are responsible for the increase in molecular stiffness. All the obtained coatings had good adhesion, high gloss, and similar yellowness, which is higher with the cationic process.

## 3. Materials and Methods

### 3.1. Materials

The following diglycidyl ethers were employed:

− Bisphenol A diglycidyl ether (DGEBA), Sigma–Aldrich (Dorset, UK) with an epoxide equivalent of 171.4 g/mol and viscosity of 5032 mPa∙s at 25 °C.
− Cyclohexane dimethanol diglycidyl ether (CHDMDE) under the trade name Grilonit^®^ V 51-63, EMS-GRILTECH (Domat/Ems, Switzerland), with an epoxide equivalent of 158.8 g/mol and viscosity of 70 mPa∙s at 25 °C.
− Neopentyl Glycol Diglycidyl Ether (NPDE), Tokyo Chemical Industry (Tokyo, Japan), characterized by the epoxide equivalent of 146.8 g/mol and viscosity of 10 mPa∙s at 25 °C.
− 1,6-Hexanediol diglycidyl ether (HDE) under the trade name Grilonit^®^ RV 1812, EMS-GRILTECH (Domat/Ems, Switzerland), characterized by the epoxide equivalent of 140 g/mol and viscosity of 7 mPa∙s at 25 °C.
− Poly (propylene glycol) diglycidyl ether (PDODE), with a molecular weight of 380 g/mol, epoxide equivalent of 158.8 g/mol, and viscosity of 41 mPa∙s at 25 °C, Sigma–Aldrich (Dorset, UK).
− Polytetrahydrofurane diglycidyl ether (PTMODE) under the trade name Grilonit^®^ F 713, EMS-GRILTECH (Domat/Ems, Switzerland), with a molecular weight of 780 g/mol, epoxide equivalent of 400.0 g/mol, and viscosity of 230 mPa∙s at 25 °C.

Methacrylic acid (MAA), 99.5% pure, hydroquinone-stabilized, was supplied by Acros Organics, Geel, Belgium. Triphenylphosphine (PPh_3_), Apollo Scientific, Bredbury, UK, was employed as a catalyst in the reaction between Ep and MAA, while hydroquinone (HQ, Acros Organics, Geel, Belgium) was employed as a polymerization inhibitor.

The following reagents and titration indicators were employed: glacial acetic acid, toluene, potassium hydroxide standard solution 0.1 M in ethanol (KOH), and crystal violet purchased from Chempur (Piekary Slaskie, Poland); chloroform from P.P.H. Stanlab (Lublin, Poland); ethyl alcohol provided by Avantor (Gliwice, Poland); tetraethylammonium bromide from Acros Organics (Geel, Belgium); perchloric acid standard solution 0.1M in glacial acetic acid supplied by Fischer Chemicals (Zurich, Switzerland); and Phenolphthalein 1% in ethyl alcohol solution from Eurochem BGD (Tarnów, Poland). All reagents were analytical grade.

### 3.2. Synthesis

**Figure 5:**
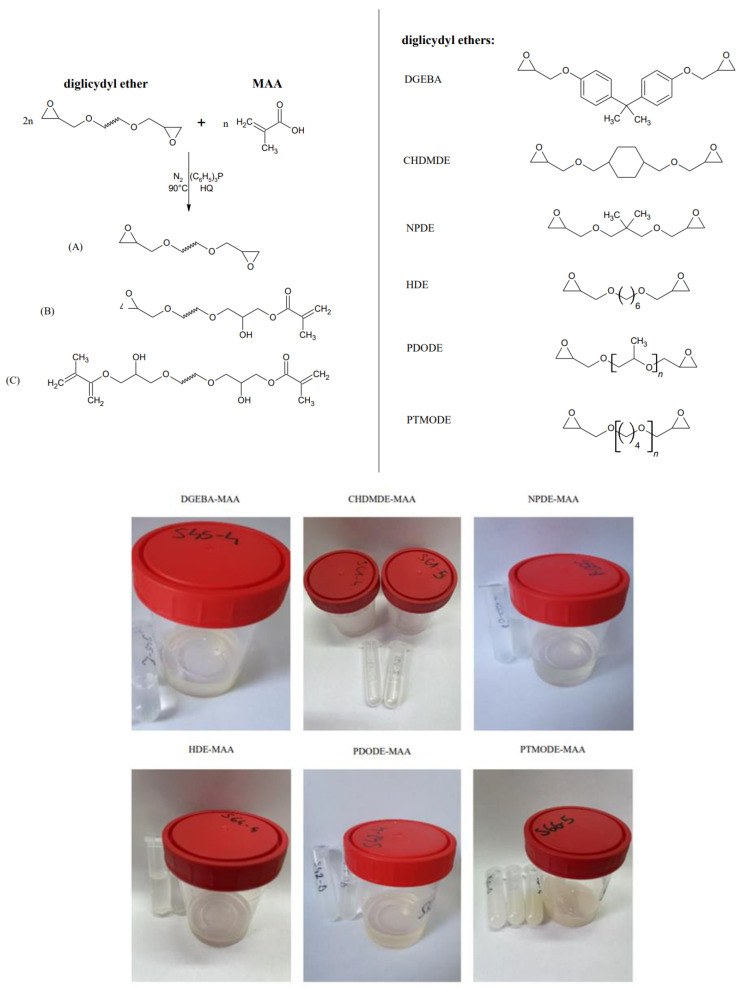
Obtaining epoxy methacrylate (diglycidyl ether-MAA) pre-polymers based on diglycidyl ethers characterized by different chain lengths/structure—general reaction scheme and product appearance.

Epoxy methacrylates (EAs) were synthesized by the addition of methacrylic acid (MAA) to epoxy resin. The process was carried out in a 250 mL glass reactor equipped with a temperature gauge, a condenser, a nitrogen inlet, and a mechanical stirrer. At the start, an appropriate amount of diglycidyl ether was introduced into the reaction vessel at room temperature; then a radical scavenger—hydroquinone—was transferred into the reactor (0.0075% by weight in relation to the total batch weight). Subsequently, methacrylic acid (0.5 mol in relation to resin epoxy value) and the catalyst (0.8% by weight in relation to the mass of MAA) were introduced. The reaction mixture was heated using an oil bath to 70 °C with stirring (120 rpm). Once the homogenous mixture was obtained, the temperature was raised to 90 °C, and the reaction was conducted for a specified time of four hours under a nitrogen atmosphere. At room temperature, the as-prepared EAs appear as colorless, transparent, viscous liquids (Figure 5). The epoxy-methacrylated pre-polymers obtained and analyzed in the present work will be denoted as DGEBA-MAA, CHDMDE-MAA, NPDE-MAA, HDE-MAA, PDODE-MAA, and PTMODE-MAA, depending on the type of diglycidyl ether used. A general pathway for the synthesis of epoxy-acrylates is presented in Figure 5.

### 3.3. Preparation of Coating Formulations and Cured Films

The coating formulations were formulated using synthesized epoxy methacrylates and 3 wt% of the photoinitiators. In the case of studying the cationic process, a cationic photoinitiator was used (bis(dodecylphenyl)iodoniumhexaflouroantimonate in propylene carbonate, Deuteron UV 1240, Deuteron, Achim, Germany). In contrast, in the case of the radical process, a radical photoinitiator (2,4,6-trimethylbenzoyl-diphenyl phosphine oxide, Omnirad TPOL from IGM resins, RM Waalwijk, The Netherlands) was used. The compositions were stirred together under dark conditions until a homogeneous mixture was employed. Then, the photocuring solution was applied to the glass substrates by means of a gap applicator (120 µm). At the end, the polymeric film was cured under a light source (UV lamp, Aktiprint-mini 18-2, type: UN50029, Technigraf GmbH, Grävenwiesbach, Germany) at room temperature and irradiated under UV light I (200 mW/cm^2^) to dryness.

### 3.4. Measurements

The FT-IR spectroscopic studies were recorded using a Thermo Nicolet 380 FT-IR spectrometer working in ATR mode (Thermo Fisher, Waltham, MA, USA). Each sample was scanned 16 times over the range of 4000–400 cm^−1^ at room temperature.

The NMR spectra were recorded in CDCl_3_ on a BRUKER DPX-400 Avance III HD spectrometer (Billerica, MA, USA) operating at 400.13 MHz (1 H) and 100.62 MHz (13 C). Tetramethylsilane (TMS) was used as the internal standard. Chemical shifts were recorded in δ (ppm), and coupling constants J were given in Hz.

The non-volatile-matter content (NV) was determined thermogravimetrically using a moisture analyzer MAX 60/NP (Radwag, Poland). The analysis was conducted at 140 °C for 30 min for the samples of approximately 1 g. The non-volatile-matter content was estimated via the following equation: NV (%) = (m_2_/m_1_) × 100%, where m_1_ is the weight of the EA sample; m_2_ is the residual weight of the sample after the measurement.

Partial acid values (PAVs) were estimated by colorimetric titration following the procedure explained earlier [27]. The methacrylic acid conversion (MAAC) was calculated via the following equation: MAAC = 100 − (PAVs∙100)/PAVs_0_, where PAVs_0_ is the initial value of PAVs (mg KOH/g).

The epoxy equivalent (EE) was determined using the colorimetric titration method [27]. The epoxy group conversion (EGC) was calculated as follows: EGC = 100 − (EE_0_ * 100)/EE_measured_ [%], where EE_0_ is the EE (g/mol) determined for diglycidyl ethers’ prior modification; EE_measured_—EE value at a specific time.

The viscosity was measured at room temperature using a cone-plate-type viscometer (LAMY REOLOGY RM-100 plus CP 2000).

The UV-curing process was isothermally monitored (25 °C) for 10 min using a photo-DSC apparatus (Q100, TA Instruments, New Castle, DE, USA) equipped with UV light emitter Omnicure S2000 (280–480 nm, 200 mW/cm^2^, Excelitas Technologies, Waltham, MA, USA). The weight of samples 15 ± 0.1 mg was placed into an open aluminum liquid DSC pan. The measurements were carried out under identical conditions. The sample was maintained at a prescribed temperature for 0.5 min before each measurement run began. The polymerization solution was composed of epoxy methacrylate resin and 3 wt% of photoinitiator. The reaction heat released in the polymerization is directly proportional to the number of epoxide (C) or methacrylate (R) groups reacted in the system. By integrating the area under the exothermic peak, the conversion of photoreactive groups or the extent of reaction was determined according to eqn: p (%) = (ΔH_t_/ΔH_0_) * 100, where ΔH_t_ is the reaction heat evolved at time t, and ΔH_0_ is the theoretical heat for complete conversion. A reaction heat (ΔH_0_) for an epoxide groups polymerization of 110 kJ mol^−1^ and methacrylate double bond polymerization of 58 kJ mol^−1^ was used. The rate of polymerization is directly related to the heat flow (dH/dt) as in the following equation: Rp (%/s) = (dH/dt)/ΔH_0_.

In order to evaluate the properties of cured coatings, the following tests were performed: tack-free time (according to ISO 9117), pendulum hardness test, cross-cut adhesion test, gloss, and yellowness index. The hardness of coatings was evaluated using a Persoz pendulum on the glass substrate (TQC Sheen, Capelle aan den IJssel, The Netherlands) according to ISO 1522 standard. The gloss was measured by means of the spectrometer method (GLS, SADT Development Technology Co. Ltd., Beijing, China) according to ASTM D523. The yellowness index was measured by means of the spectrometer method according to ASTM E313 using a precision colorimeter NH-145 (3NH Technology Co. Ltd., Shenzhen, China).

## 4. Conclusions

In this article, the syntheses of six epoxy methacrylic compounds were made based on diglycidyl ethers of various structures (linear, aliphatic, or aromatic rings, with a shorter or polymeric backbone) were presented. The synthesis’s essence was adding methacrylic acid to epoxy groups of ethers in such a way that hybrid structures, containing both epoxy groups and double bonds, remained in the post-reaction mixture. The modification progress was monitored by titration (epoxy equivalent and acid number), and the structure of the expected products was validated by infrared spectroscopy and nuclear magnetic resonance. The conducted modification allowed us to obtain products that can polymerize both cationic and radical mechanisms. Moreover, the obtained products (apart from the compound based on bisphenol A diglycidyl ether—DGEBA) are characterized by low viscosity (100–500 Pa∙s), which makes them extremely useful for the production of coating materials. Hence, the research presents the influence of different chain architectures of synthesized epoxy methacrylate resins (EAs) based on low-viscous diglycidyl ethers on the course of the photocuring process according to the cationic and radical mechanisms. It was shown that the epoxy methacrylate pre-polymers with shorter chain lengths and features of chemical structure such as aromatic, cycloaliphatic, or some branched structures, i.e., DGEBA, CHDMDE, and NPDE, polymerize faster and obtain higher conversion of epoxy or methacrylic groups in contrast to epoxy methacrylates based on diglycidyl ethers containing a linear carbon chain structure, i.e., HDE, PDODE, and PTMODE. The obtained cured coatings have a wide range of properties, which makes them potentially valuable for applications as protective coatings, varnishes, or films in various industries.

## Figures and Tables

**Table 1:** Characteristic of manufactured epoxy methacrylate pre-polymers.

Sample Code	NV (%)	PAVs (mg KOH/g)	MAAC (%)	EE (g/mol)	EGC (%)	η (mPa·s)
DGEBA-MAA	98.53	10.2	93.5	312.753	59.1	21320
CHDMDE-MAA	95.11	26.1	88.3	250.675	36.7	487
NPDE-MAA	88.10	50.2	85.1	298.973	50.9	317
HDE-MAA	96.63	4.2	97.8	258.150	45.7	104
PDODE-MAA	94.13	32.9	77.0	288.651	35.4	203
PTMODE-MAA	98.43	9.3	86.8	676.633	40.9	397

NV—non-volatile-matter content; PAVs—partial acid numbers; MAAC—methacrylic acid conversion; EE—epoxy equivalent; EGC—epoxy group conversion; η—viscosity.

**Table 2:** Molar ratios of relevant epoxy methacrylate pre-polymers.

Sample Code	n—Number of Polymer Segments	Molar Ratios of Individual Products		
		A	B	C
DGEBA-MAA	1	0.94	1.00	0.83
CHDMDE-MAA	1	0.00	1.00	0.00
NPDE-MAA	1	0.00	1.00	1.08
HDE-MAA	1	1.20	1.00	1.13
PDODE-MAA	3	0.00	1.00	0.11
PTMODE-MAA	9	0.22	1.00	0.00

A—neat diglycidyl ether; B—hybrid compound having both epoxy and vinyl end-groups; C—a compound with vinyl-end groups.

**Table 3:** Characteristic of the photocuring process of epoxy methacrylate pre-polymers.

Sample Code	ΔH_total_ (J/g)		t_max_ (s)		p_max_ (%)		Rp_max_ (%/s)	
	C	R	C	R	C	R	C	R
DGEBA-MAA	138.0	163.7	0.07	0.07	25	45	32.53	58.40
CHDMDE-MAA	325.4	400.8	0.26	0.05	33	49	31.79	65.54
NPDE-MAA	284.8	506	0.22	0.03	18	76	18.97	118.13
HDE-MAA	98.6	120.5	0.10	0.05	6	23	5.63	29.72
PDODE-MAA	107.8	120.3	0.21	0.20	4	10	5.22	8.95
PTMODE-MAA	23.2	28.0	0.02	0.01	3	4	4.36	4.38

ΔH_total_—total enthalpy of the photocuring process; t_max_—time to achieve maximum heat flow (the time when there was no radiation subtracted); p_max_—maximum conversion degree of the epoxide groups (for C) or acrylate double bonds (for R); Rp_max_—maximum rate of conversions.

**Table 4:** Properties of the cured coatings prepared with obtained epoxy methacrylates depend on the photopolymerization mechanism (C—cationic, R—radical).

Sample Code	Tack-Free Time (s)		Hardness (s)		Adhesion		Gloss (GU)		Yellowness Index	
	C	R	C	R	C	R	C	R	C	R
DGEBA-MAA	15	9	143	161	2.5	2	165	160	4.2	4.1
CHDMDE-MAA	90	21	108	120	1	1	148	154	7.3	6.9
NPDE-MAA	90	90	66	67	2	2.5	180	64	3.9	3.0
HDE-MAA	90	90	54	63	1	2.5	134	130	8.3	7.3
PDODE-MAA	90	90	32	52	1	2.5	150	52	5.1	3.4
PTMODE-MAA	90	-	30	-	3.5	-	150	-	7.3	-

No curing.

## Data Availability

Not applicable.

## Supplementary Materials

The following supporting information can be downloaded at: https://www.mdpi.com/article/10.3390/ijms232415592/s1.
