# Supplementary material for: Synthesis of Hybrid Epoxy Methacrylate Resin Based on Diglycidyl Ethers and Coatings Preparation via Cationic and Free-Radical Photopolymerization
Source: Int J Mol Sci. 2022 Dec 9;23(24):15592. doi: 10.3390/ijms232415592 (PMC9779419; doi:10.3390/ijms232415592)
Supplement: Supplementary file 1 [file ijms-23-15592-s001.zip › ijms-2040833-supplementary.pdf]

# Supplementary data

## Synthesis of Hybrid Epoxy Methacrylate Resin Based on Diglicydyl Ethers and Coatings Preparation *via* Cationic and Free-Radical Photopolymerization

Paulina Bednarczyk[a*], Izabela Irska[b], Konrad Gziut[a], Karolina Mozelewska[a], Paula Ossowicz-Rupniewska[a]

[a] West Pomeranian University of Technology in Szczecin, Faculty of Chemical Technology and Engineering, Department of Chemical Organic Technology and Polymeric Materials, Piastów Ave. 42, 71-065 Szczecin, Poland
[b] West Pomeranian University of Technology in Szczecin, Faculty of Mechanical Engineering and Mechatronics, Department of Materials Technology, Piastów 19 Avenue, 70-310 Szczecin, Poland

*Corresponding author: Paulina Bednarczyk e-mail: bednarczyk.pb@gmail.com, paulina.bednarczyk@zut.edu.pl

---

Number of pages: 15
Number of Figures: 14

## Table of Contents

1. The NMR spectra of substrates and epoxy methacrylated pre-polymers S2-S15

## The NMR spectra of substrates and epoxy methacrylated pre-polymers

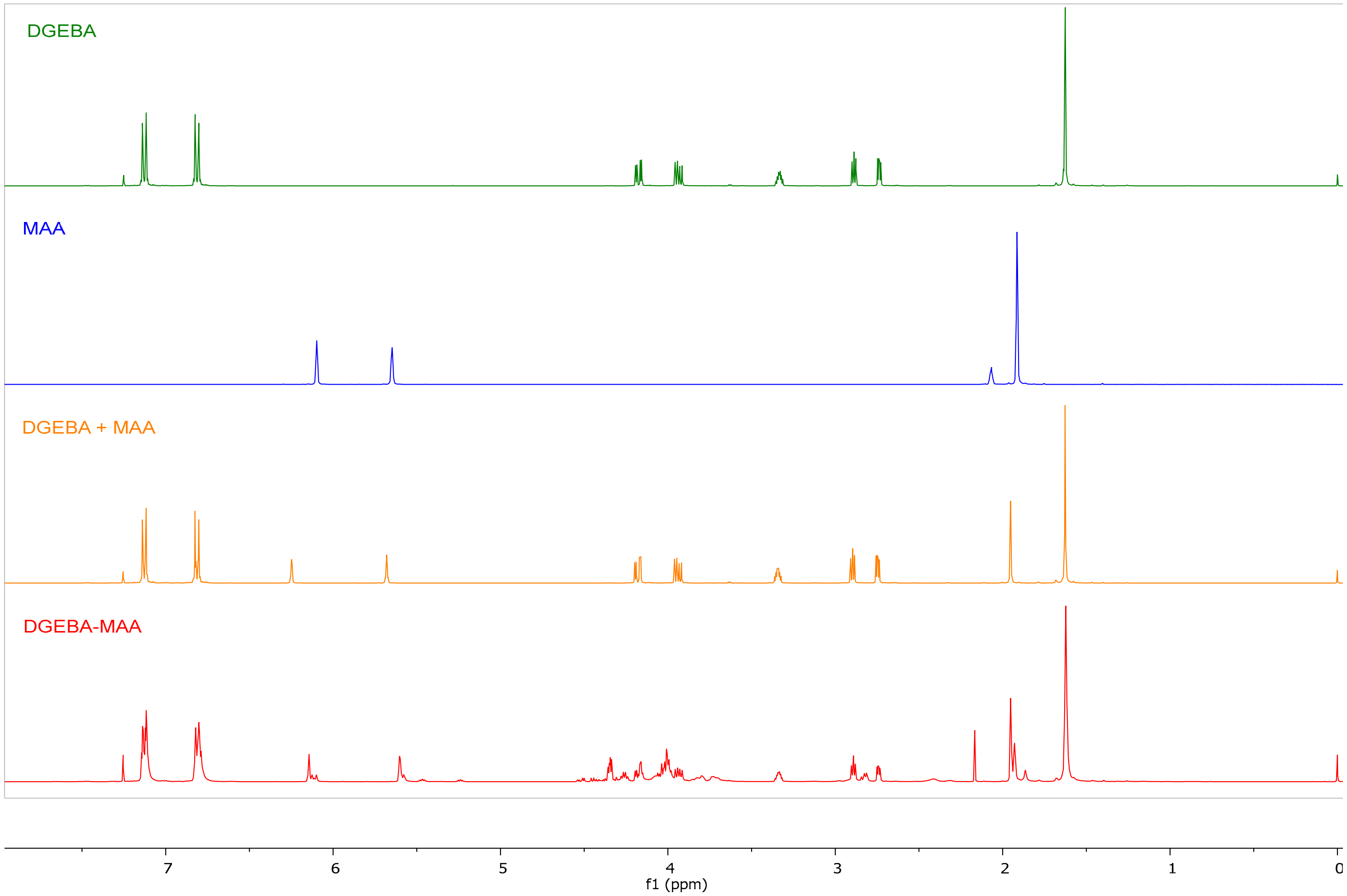


**Figure S1.** $^1$H NMR spectra of DGBA (green), MAA (blue), the mixture of DGBA and MAA (orange), and reaction mixture DGBA-MAA (red).

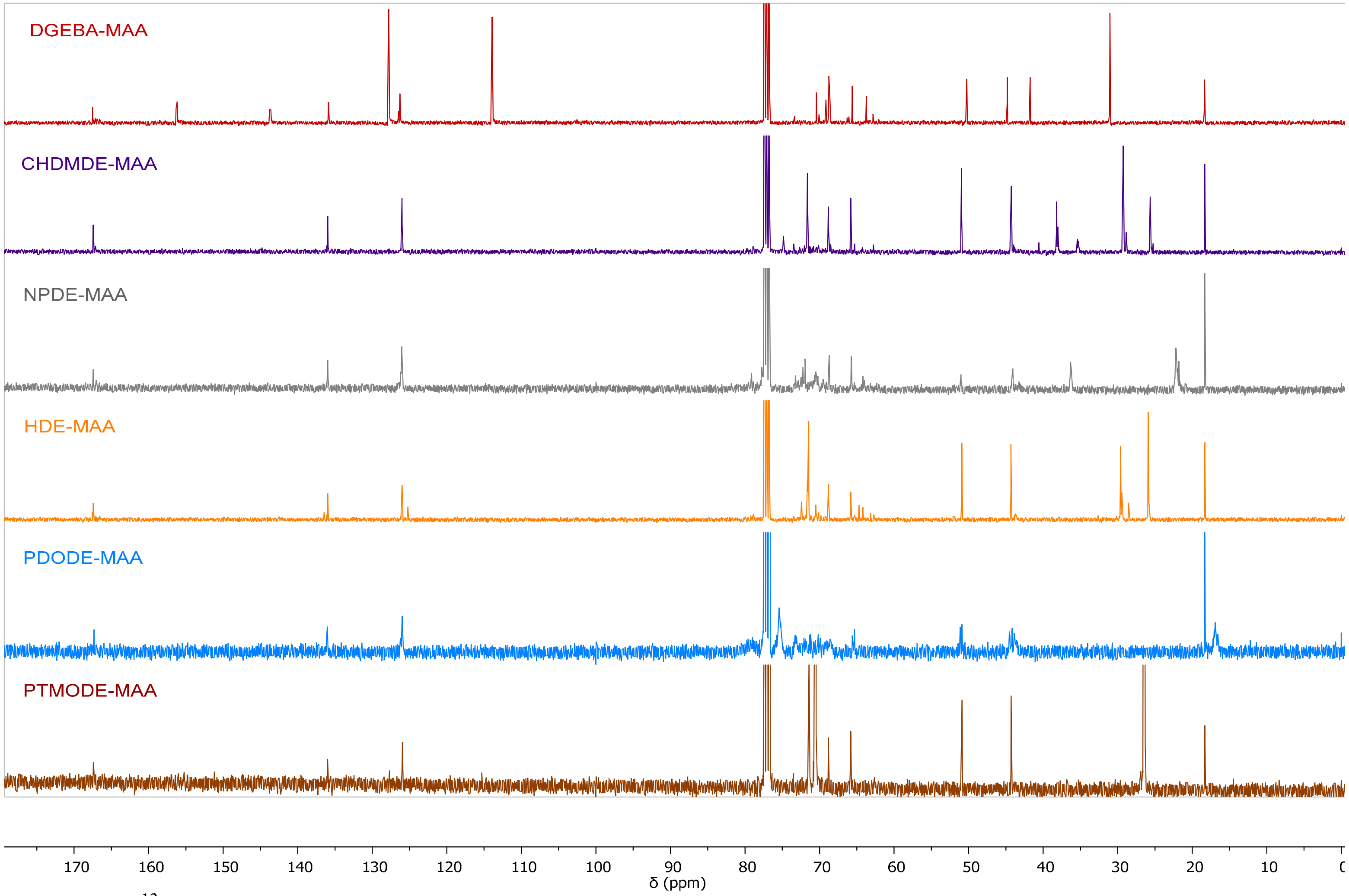


**Figure S2.** $^{13}$C NMR spectra of DGBA-MAA (red), CHDMDE-MAA (violet), NPDE-MAA (grey), HDE-MAA (orange), PDODE-MAA (blue), PTMODE-MAA (brown).

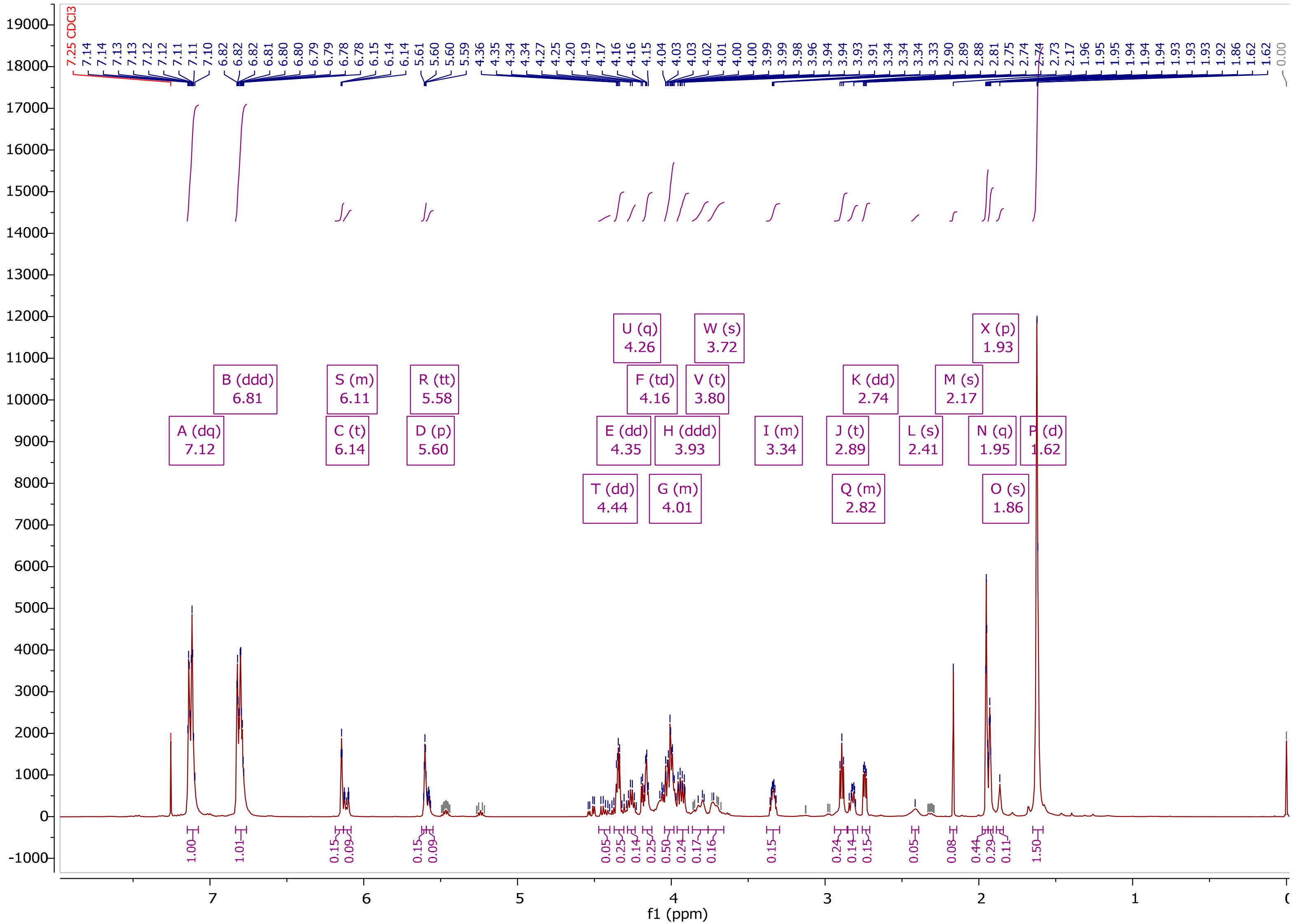

**Figure S3.** $^{1}$H NMR spectra of reaction mixture DGBA-MAA.

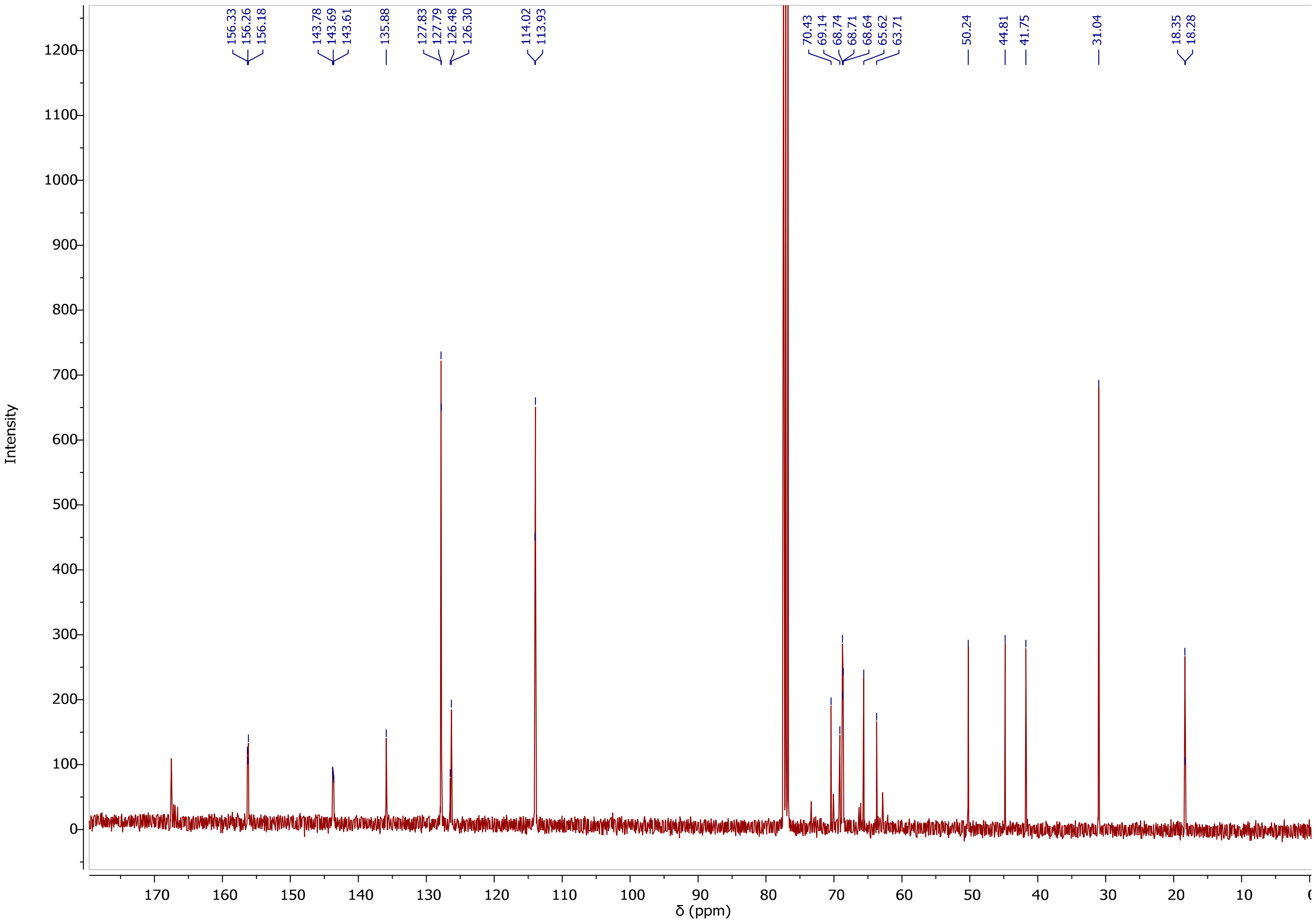


**Figure S4.** $^{13}$C NMR spectra of reaction mixture DGBA-MAA.

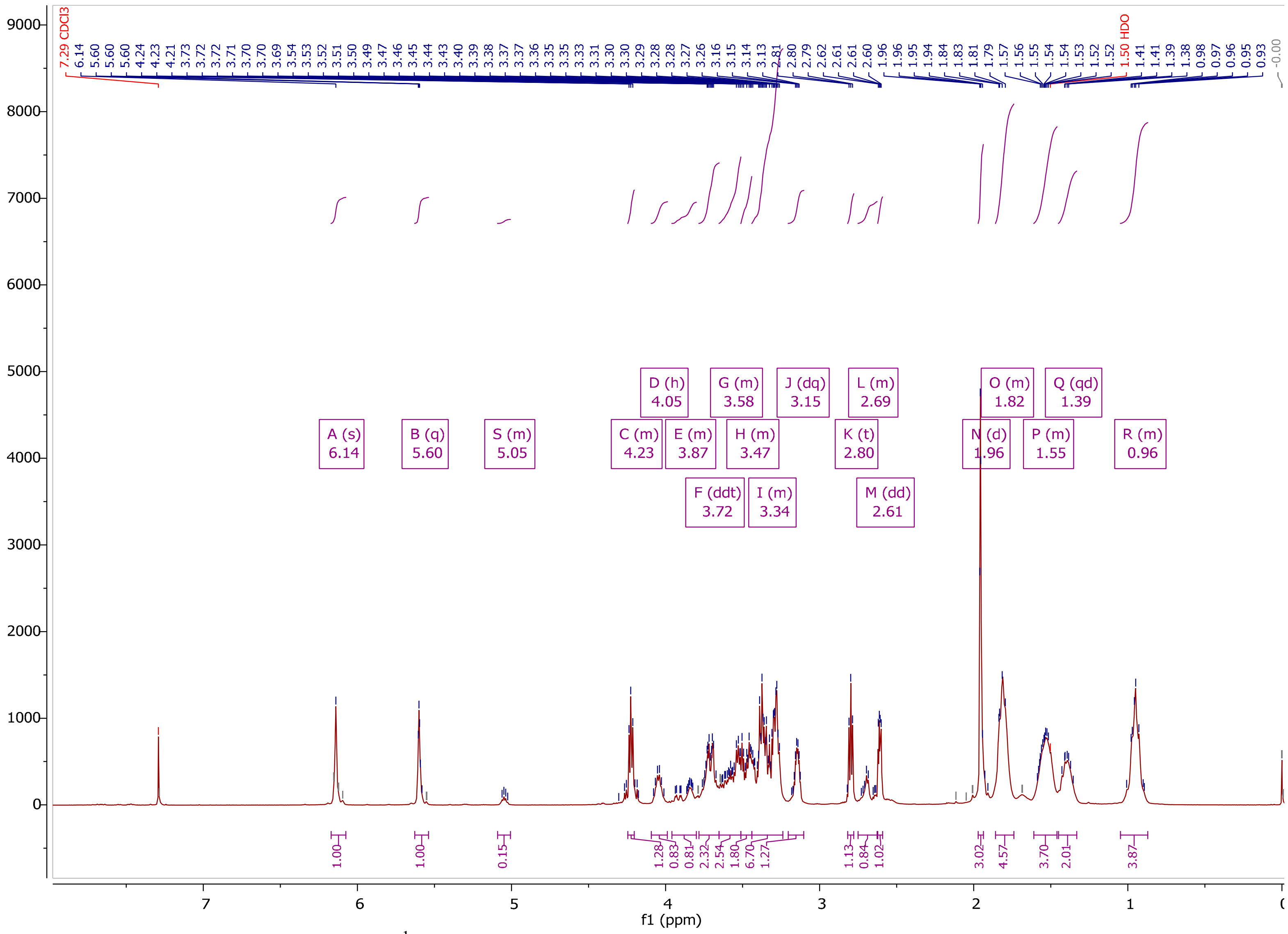


**Figure S5.** $^{1}$H NMR spectra of reaction mixture CHDMDE-MAA.

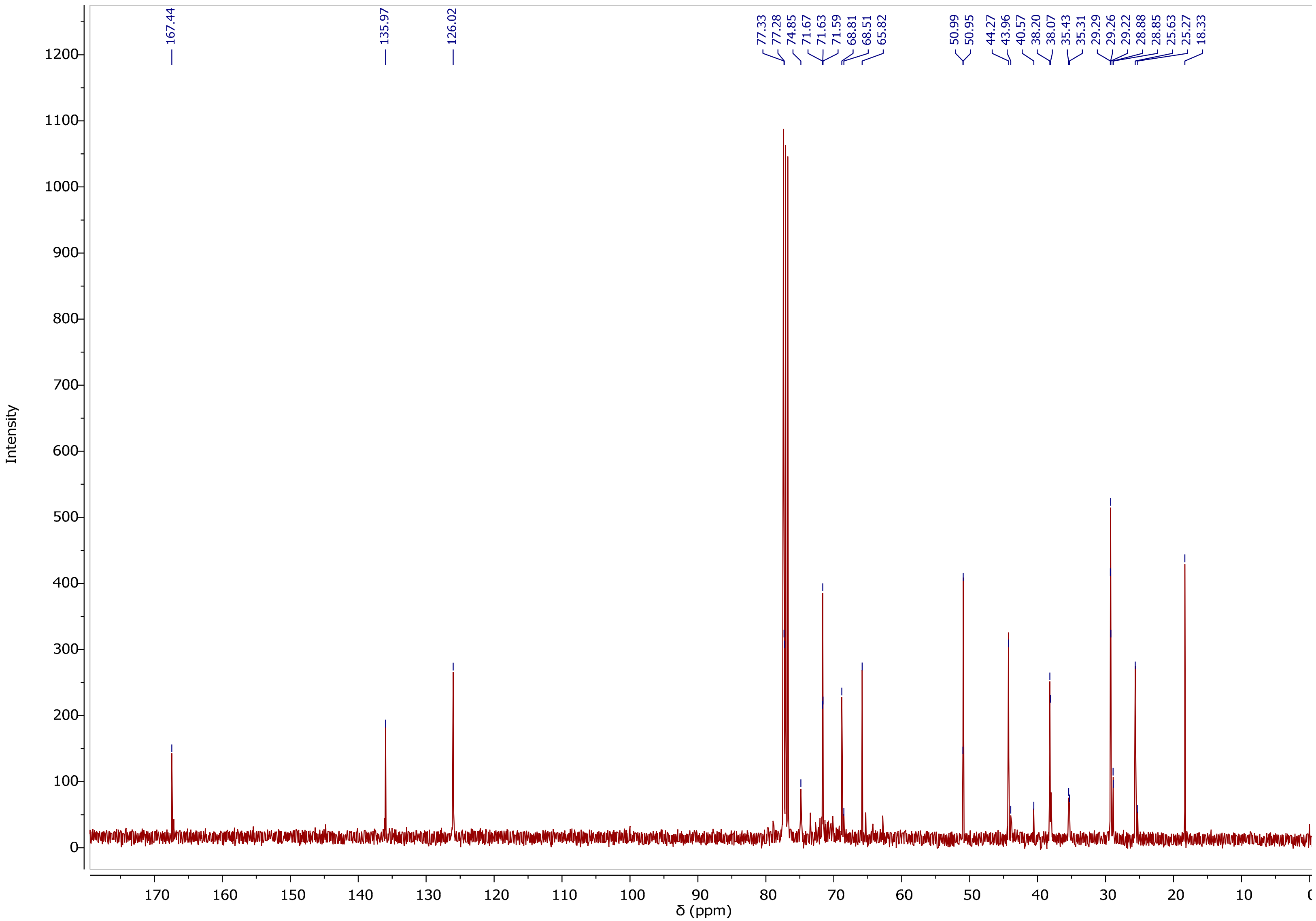


**Figure S6.** $^{13}$C NMR spectra of reaction mixture CHDMDE -MAA.

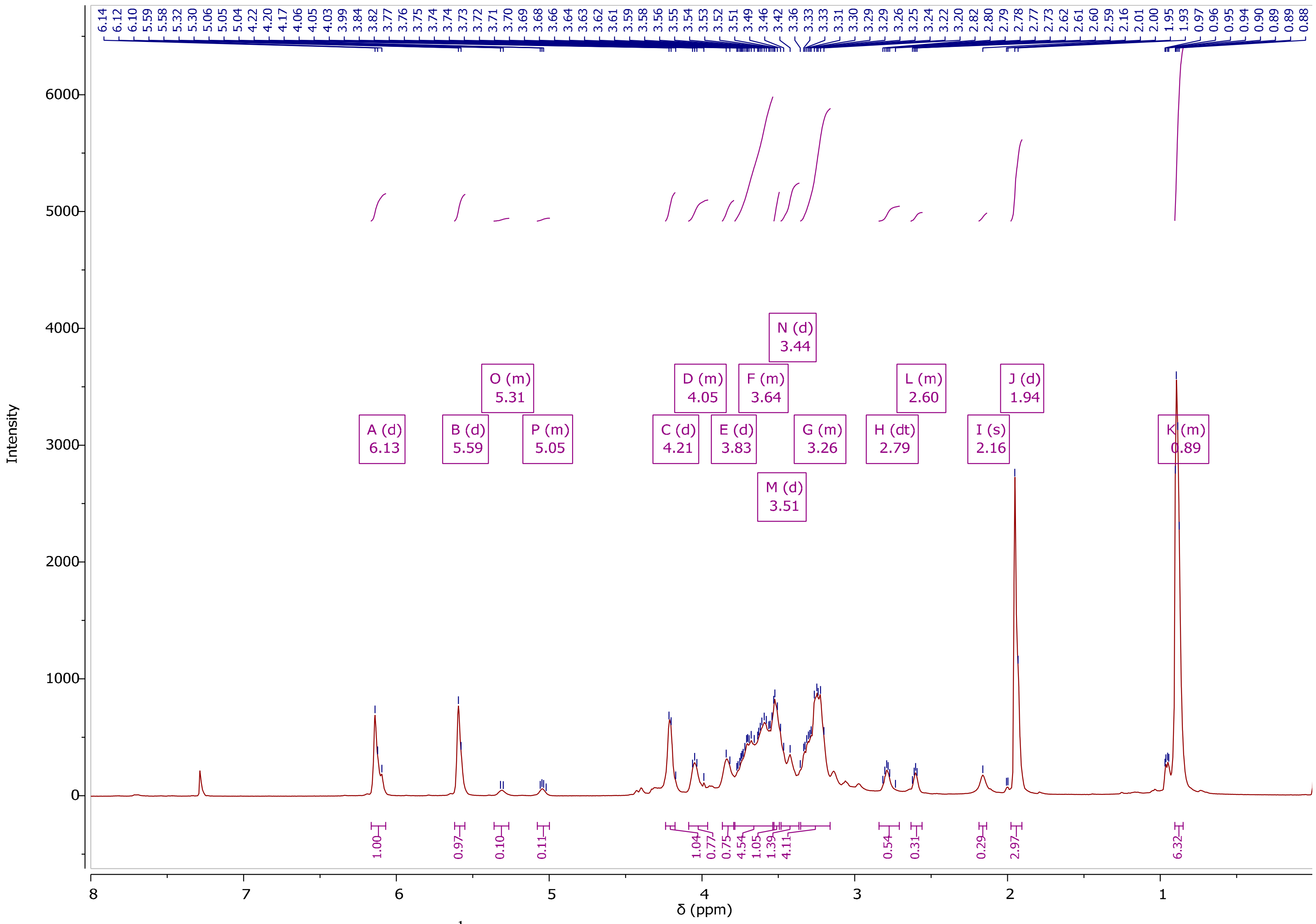


**Figure S7.** $^{1}H$ NMR spectra of reaction mixture NPDE-MAA.

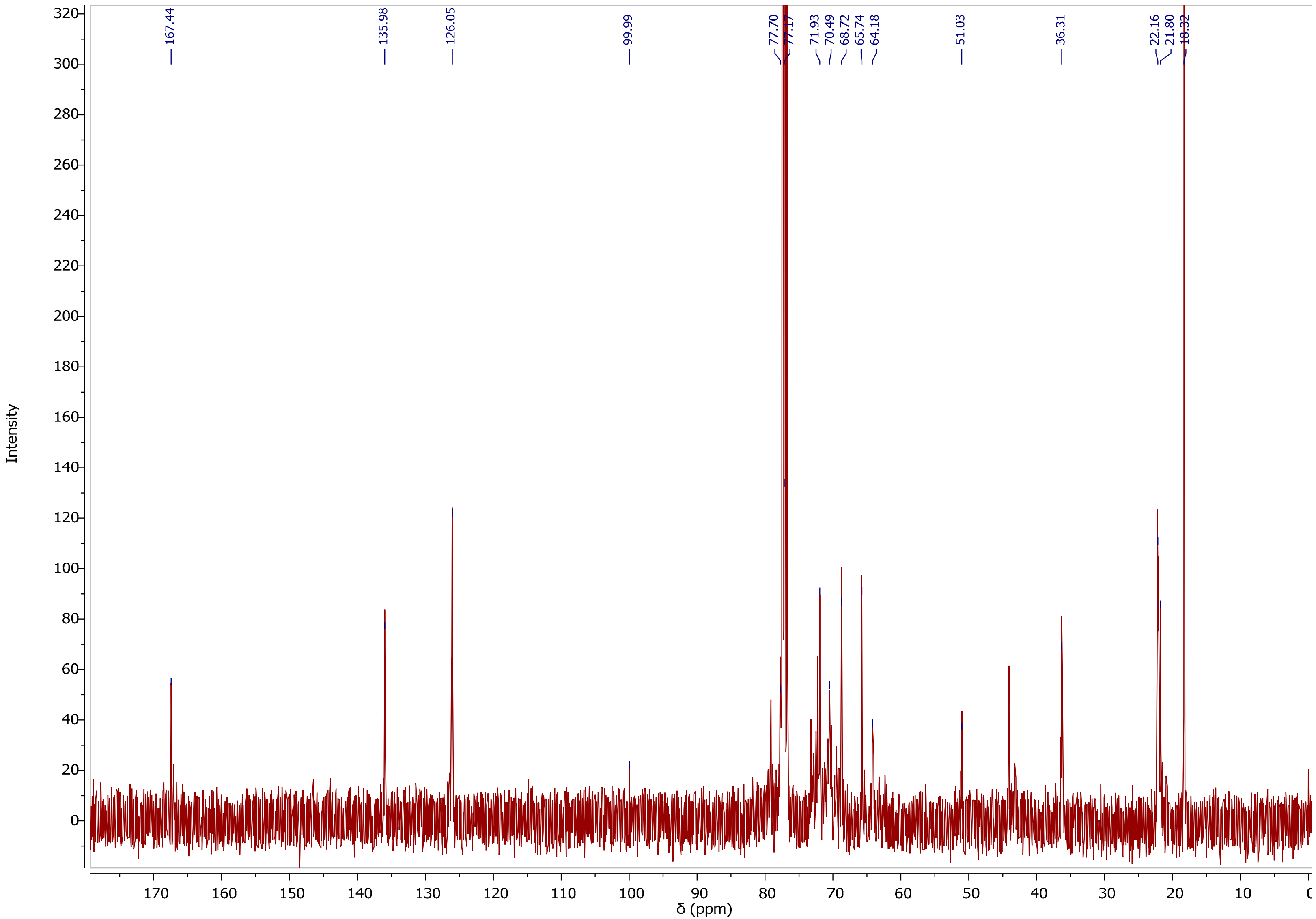


**Figure S8.** $^{13}C$ NMR spectra of reaction mixture NPDE -MAA.

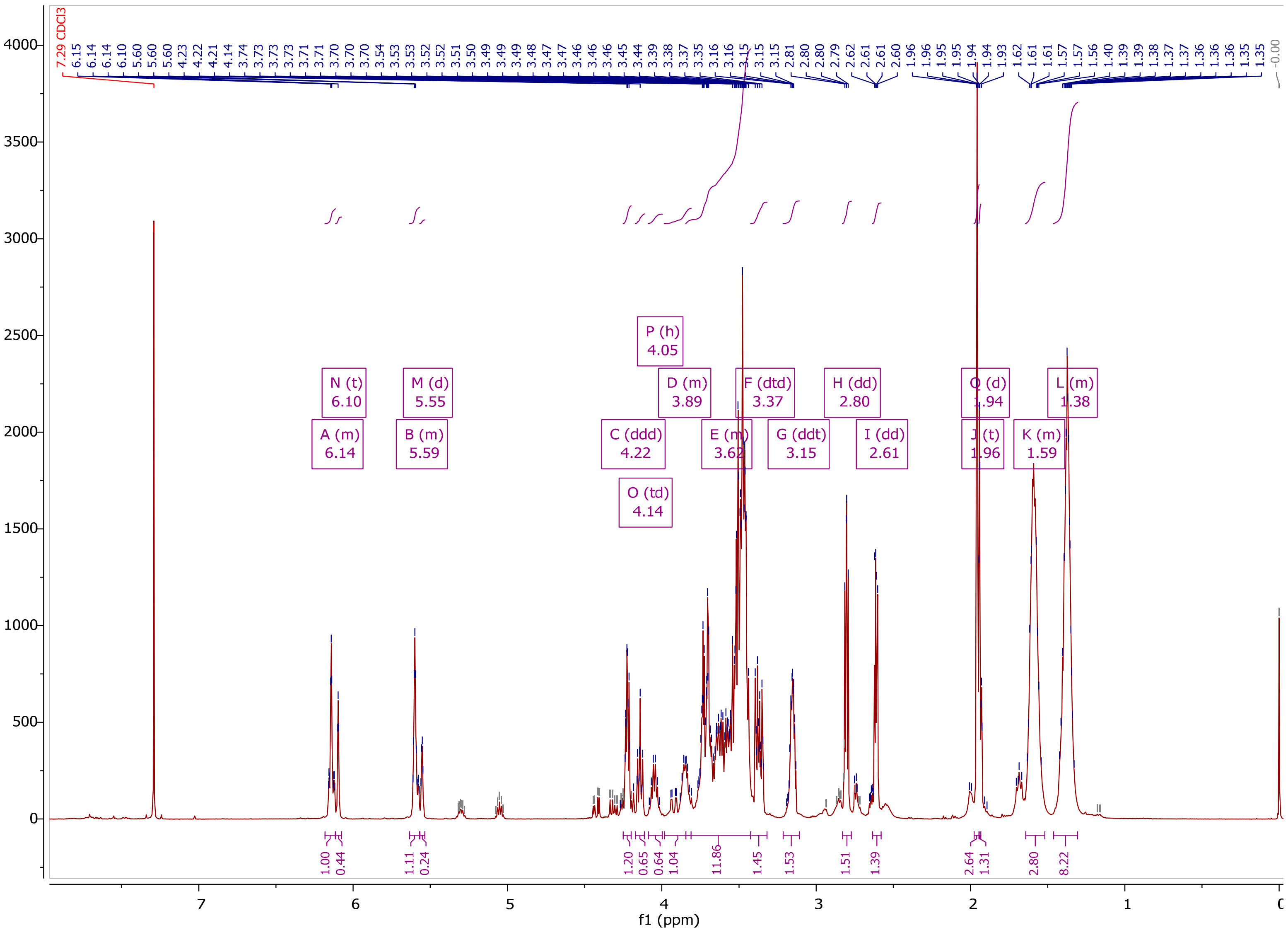


**Figure S9.** $^1$H NMR spectra of reaction mixture HDE-MAA.

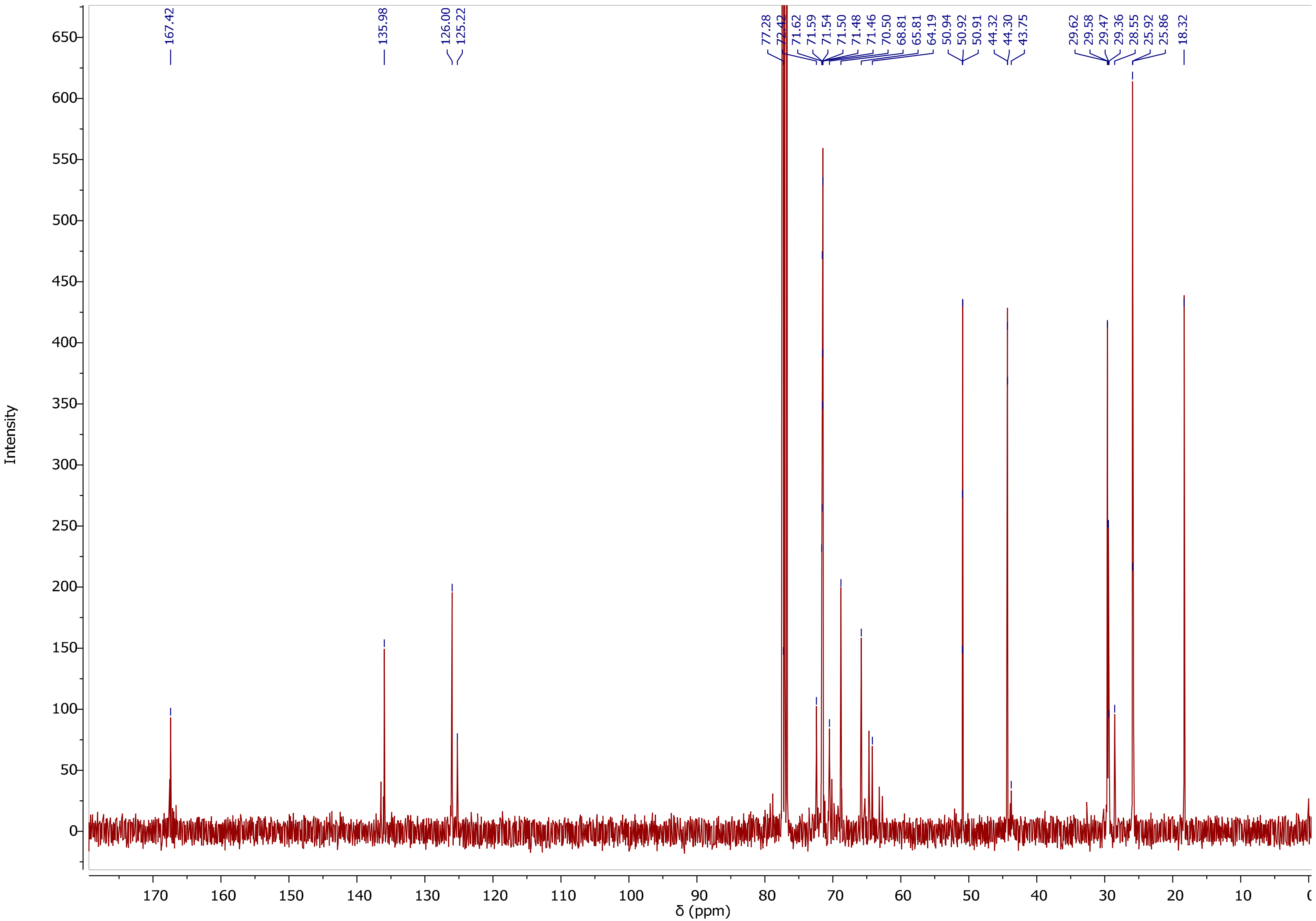

**Figure S10.** $^{13}$C NMR spectra of reaction mixture HDE -MAA.

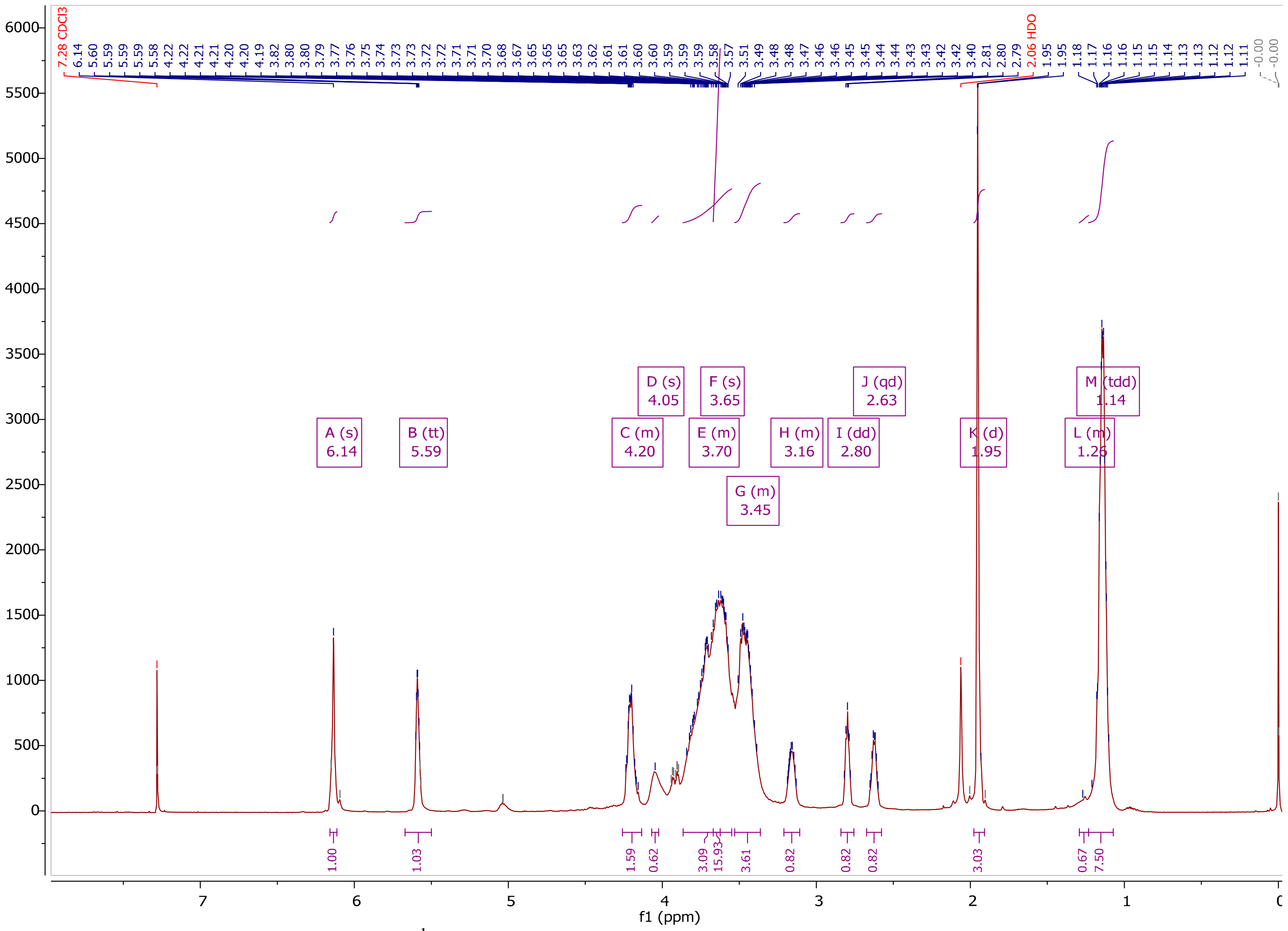


**Figure S11.** $^1$H NMR spectra of reaction mixture PDODE-MAA.

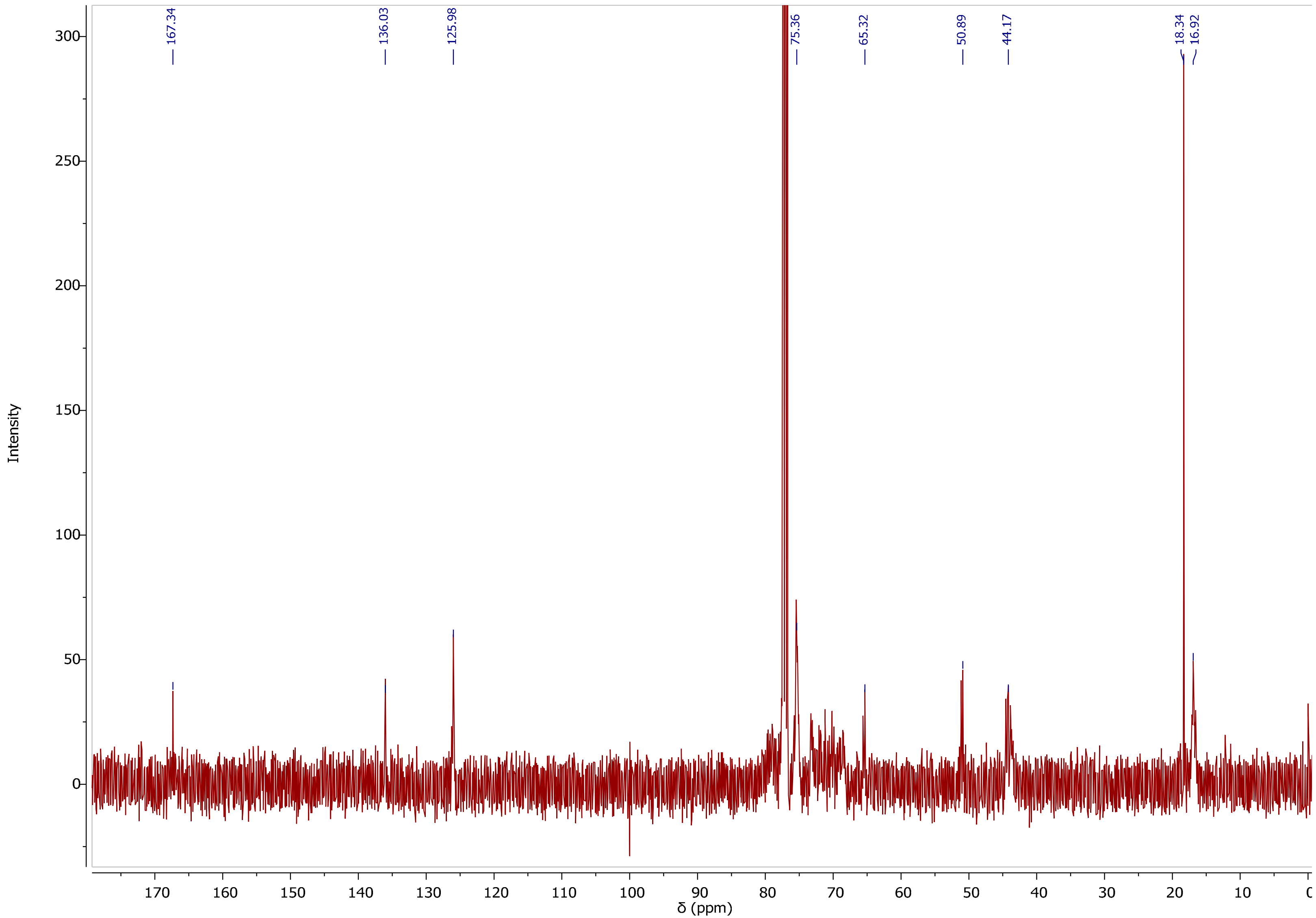


**Figure S12.** $^{13}$C NMR spectra of reaction mixture PDODE -MAA.

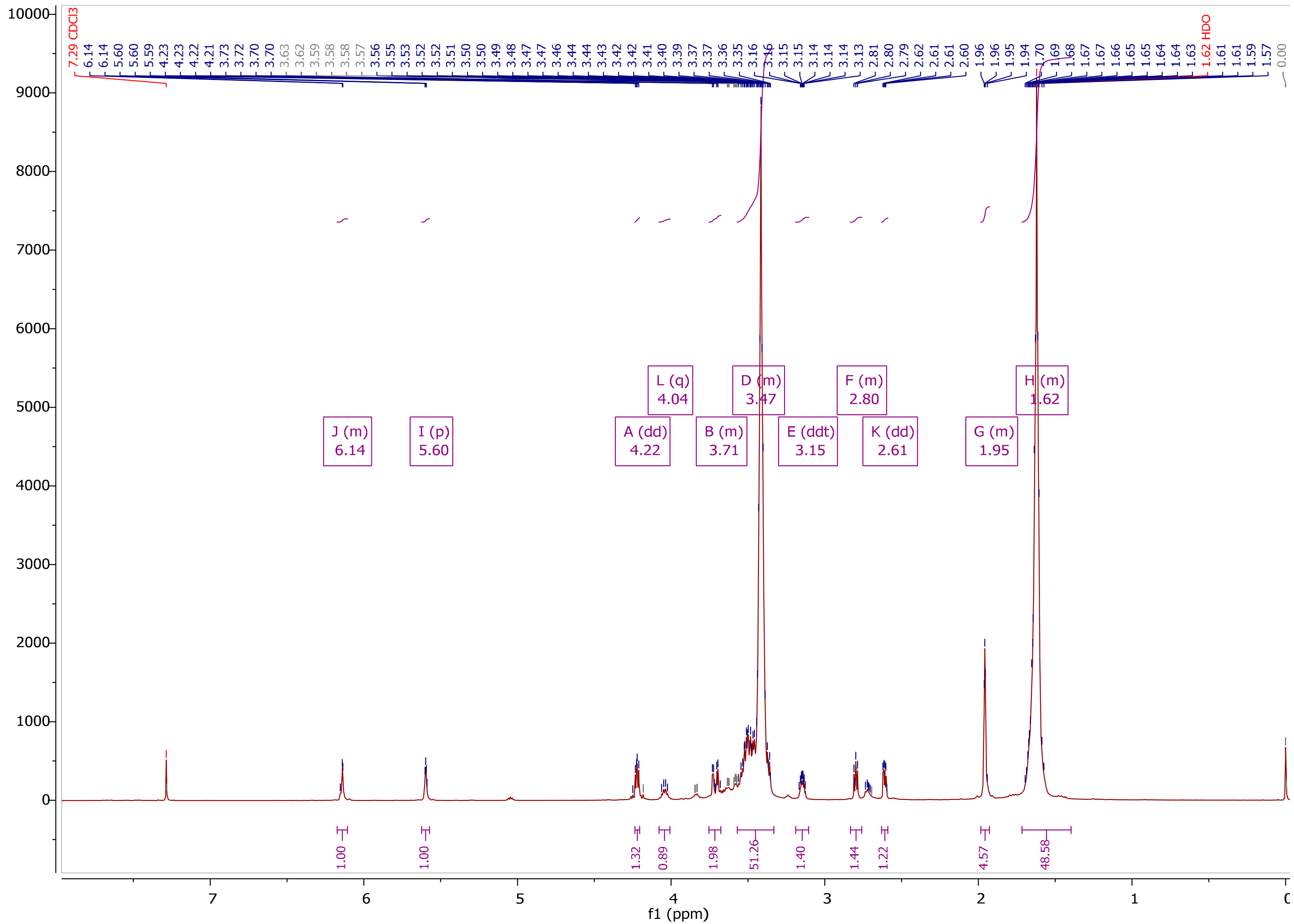


**Figure S13.** $^1$H NMR spectra of reaction mixture PTMODE-MAA.

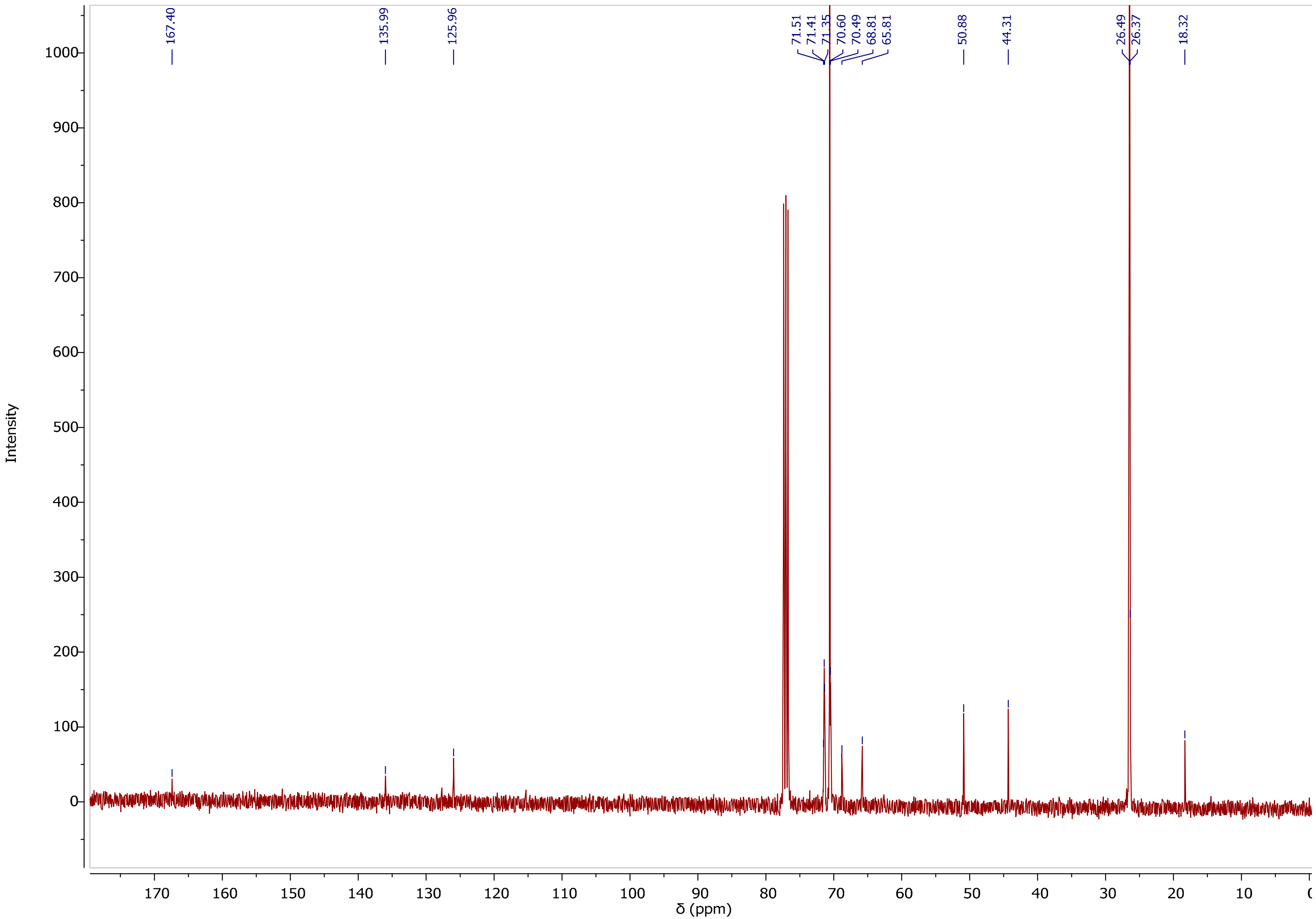


**Figure S14.** $^{13}$C NMR spectra of reaction mixture PTMODE -MAA.
